# Development, delivery, and evaluation of a training program for the early identification of autism: Monitoring of Social Attention, Interaction, and Communication

**DOI:** 10.3389/fneur.2023.1201265

**Published:** 2023-07-07

**Authors:** Melissa Gilbert, Katherine Gore, Marguerite Hawke, Josephine Barbaro

**Affiliations:** Olga Tennison Autism Research Centre, School of Psychology and Public Health, La Trobe University, Bundoora, VIC, Australia

**Keywords:** autism, early identification, maternal and child health nurses, public health, workplace training

## Abstract

**Introduction:**

Early identification of Autistic children is an important precursor to diagnosis, and access to supports and services. Here we describe the training of the maternal and child health (MCH) workforce in the state of Victoria, Australia in the early identification of infants and toddlers with a high likelihood of autism.

**Methods:**

In 2019, 1,428 MCH nurses completed early autism training held at venues across the state, with an additional 82 nurses completing online-only training. A training needs analysis enabled the research team to determine the workforce’s current skill and knowledge levels, and to identify knowledge gaps, training needs and workplace barriers. The professional development program, known as Monitoring of Social Attention, Interaction, and Communication (MoSAIC), comprised: online pre-workshop modules; a face-to-face instructor-led workshop, which included the use of the Social Attention and Communication-Revised (SACS-R) tool; and online post-workshop modules, which included a recording of a face-to-face workshop with all accompanying resources. This was the first time that the MCH workforce received this training package. Attendees were asked to complete a training satisfaction survey immediately following the face-to-face instructor-led workshop and a follow-up survey regarding their autism knowledge and SACS-R implementation 4–6 weeks after the workshop.

**Results:**

Over 90% (*n* = 325) of MCH nurses who completed the training satisfaction survey agreed or strongly agreed with statements that the training was clear and of high quality. Most nurses also reported that the training was well-presented and that they would recommend it to a colleague. In the 6 months following the training, a total of 82,581 SACS-R assessments were conducted by the MCH workforce, reflecting that MCH nurses had successfully integrated SACS-R assessments into their work practice after receiving the early autism identification training.

**Discussion:**

This study demonstrated that training on the early identification of autism can be successfully designed, customized, and delivered to a large primary healthcare workforce for universal developmental surveillance of autism.

## 1. Introduction

In Australia, professionals such as maternal and child health (MCH) nurses, general practitioners (GPs; or family doctors), pediatricians, speech pathologists, psychologists, and early childhood educators are involved in the early identification of children with a high likelihood of autism (1). The early identification of children on the autism spectrum is critical, as parents/caregivers usually only seek diagnosis and supports after becoming aware their child has additional support needs (2). In accordance with the Australian National Guideline for Autism Assessment (3), an autism diagnosis can be made by pediatricians, psychiatrists, neurologists, clinical or educational/developmental psychologists, or neuropsychologists as a single clinician diagnosis. Alternatively, the clinician may seek consensus from a multidisciplinary team that may include occupational therapists, speech pathologists, or other psychologists. Early identification of Autistic children is crucial as is it shown to reduce family stress (4), as well as lowering the need for ongoing support for children and families (5), as well as reducing ongoing costs across the lifespan (6). The delay between concern and diagnosis (7) is a barrier to accessing early supports and services, which are known to enhance cognitive, emotional, and adaptive outcomes for Autistic children (5, 8–10).

MCH nurses are well placed to be involved in the early identification and support of Autistic^1^ infants and toddlers. The MCH service in the state of Victoria, Australia is free and universally available to all children residing in the state. MCH nurses monitor children’s health and developmental milestones, providing parents/caregivers with guidance relevant for each age range, and referring families to other services where warranted (11). Parents/caregivers and their children attend regular ‘key ages and stages’ (KAS) appointments with an MCH nurse, with 10 KAS appointments scheduled from birth to 42 months of age, with *ad hoc* appointments added if required (12). At 2 weeks of age, 96.7% of infants residing in Victoria and their parents/caregivers access the MCH service, with attendance remaining above 70% across KAS appointments until 24 months of age (13). Similar services with high utilization are found both nationally (14) and internationally (15, 16).

Previous studies in the United States (17), Japan (18), and the United Kingdom (19) have demonstrated MCH nurse capability in the early identification of autism. Similarly, two studies from Victoria, Australia have evaluated training for MCH nurses in key social communication milestones and the use of the Social Attention and Communication Surveillance [SACS; (20, 21)] early autism identification tool and its revised version, the Social Attention and Communication Surveillance-Revised [SACS-R; (22)]. In these studies, MCH nurses used the observationally based items in the SACS-R 12-, 18-, and 24-month checklists to determine community-based children’s likelihood of autism. Each checklist comprised 12 to 15 age-appropriate social communication behavioral items that can be infrequent, inconsistent, or absent in Autistic infants and toddlers. These studies have demonstrated that SACS/SACS-R tools are the most accurate population-based early autism screening tools, with high diagnostic accuracy (positive predictive value >83%; SACS-R estimated negative predictive value = 99%) (21, 22, 25). Additionally, the SACS-R has a much lower mean age of identification (21.2 months) (22) compared to the current mean age of diagnosis in Australia (49.2 months) (26).

In September 2018, the Victorian Government committed funding to train all MCH nurses working in the state of Victoria on the early identification of autism and the use of SACS-R tool (27). The study team sought to gain a better understanding of existing MCH nurse competencies in early autism identification to develop an effective, targeted professional development program (PDP) called Monitoring of Social Attention, Interaction and Communication (MoSAIC), for a large workforce. In this paper, we describe the process of the training needs analysis (TNA), training delivery, and analysis of MCH nurse training satisfaction. The aim of this study was to develop a PDP for the MCH workforce on the early signs of autism, social communication milestones, and the use of the SACS-R tool that considered the needs of a wide range of stakeholders.

## 2. Materials and methods

**Figure 1:**
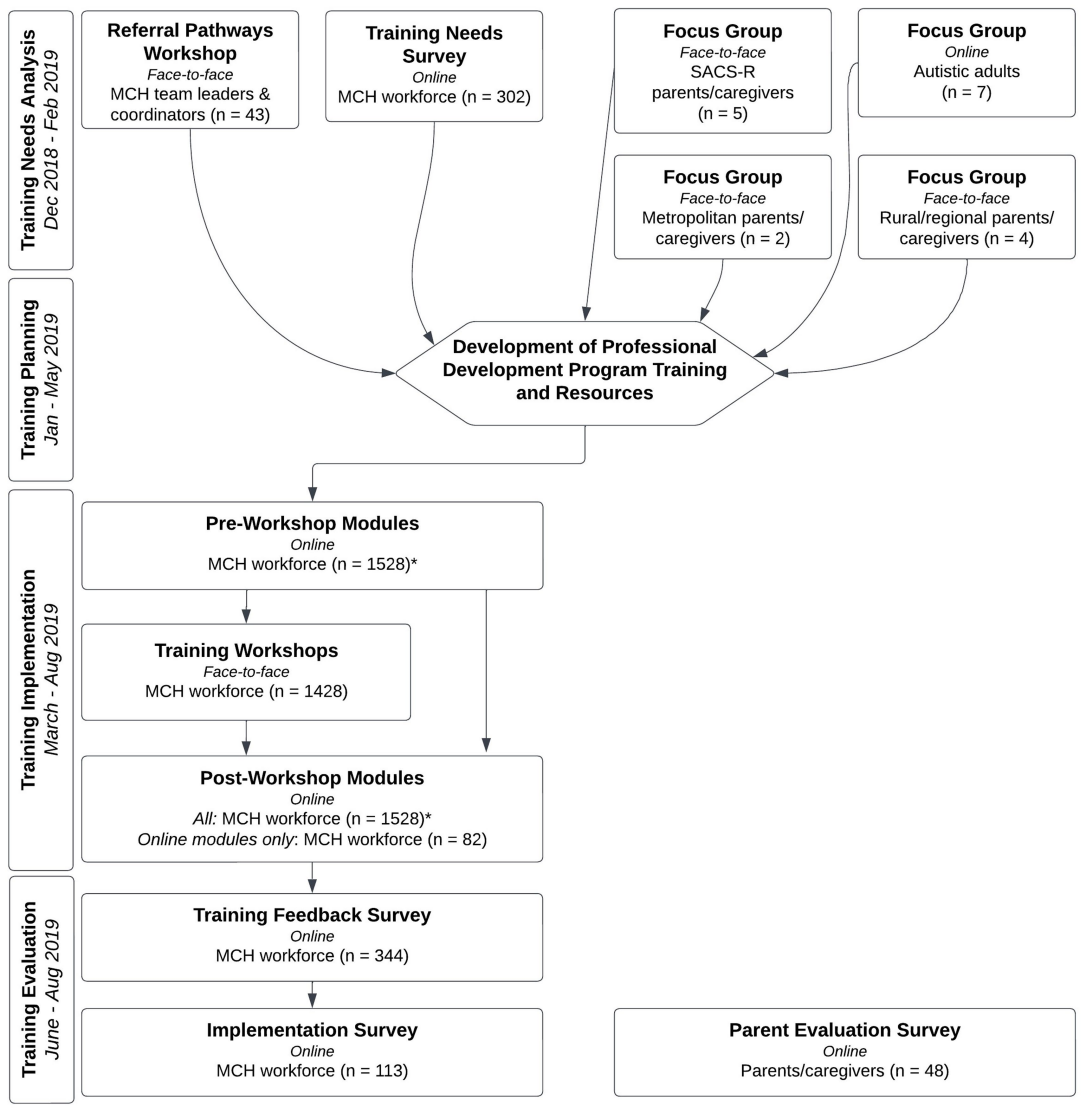
Maternal and child health workforce early autism identification professional development program flowchart.

The PDP discussed here was comprised of four components: a training needs analysis; training planning; training implementation; and training evaluation (Figure 1). Each element is outlined sequentially below.

### 2.1. Ethics approvals

Approval for this study was gained from the La Trobe University Human Ethics Committee (HEC18463 and HEC19042) and the Department of Education and Training (DET; 2018_003864).

### 2.2. Training needs analysis

A broad approach was taken when conducting the TNA, enabling the research team to determine MCH nurse training needs from a variety of stakeholders, including member of the MCH workforce, Autistic adults, and parents/caregivers. This was comprised of: (1) a referral pathways workshop; (2) focus groups; and (3) a training needs survey [(28); Figure 1]. All online participant information and consent forms and questionnaires in the TNA were hosted on the Qualtrics online survey platform.

#### 2.2.1. Referral pathways workshop

A workshop was held on 30 November 2018 with MCH nurse team leaders and coordinators to identify the current referral pathways used for children identified with a high likelihood for autism; barriers experienced; feedback on a referral pathway proposed by the research team; and useful referral and support pathway resources and information. At the start of the workshop participants ranked the referral services to which children with a high likelihood of autism were commonly referred [General Practitioner (GP) Practitioner (GP); Early Childhood Early Intervention (ECEI); Early Childhood Intervention Services (ECIS); Community Allied Health; and Private Allied Health] in order of whom they most commonly referred to. GPs were included on this list due to a referral from a GP being required to access the medical practitioners qualified to diagnose autism in Australia, with MCH nurses unable to make these referrals directly. The remaining services were included as they were the primary sources of supports and services for young Autistic children. Following this, a discussion of referral pathways, supports, and services for children identified with a high likelihood of autism was led by the research team. The group was then split into two – one group of participants from the metropolitan area and another group of participants from rural/regional areas. The break-out sessions provided participants with the opportunity to further discuss location-specific issues and barriers. The groups reunited to discuss and refine the referral pathway proposed by the research team, for children identified with a high likelihood of autism.

Participant selection criteria were that participants were over the age of 18 and were currently employed as a MCH nurse team leader or coordinator in Victoria. All Victorian MCH nurse team leaders and coordinators were invited by email to participate in the workshop, with a link to the online participant information and consent form included in the email. A total of 43 MCH nurse team leaders and coordinators participated in the workshop.

#### 2.2.2. Focus groups

Four focus groups were conducted as part of the TNA: (1) metropolitan parents/caregivers, (2) rural/regional parents/caregivers, (3) SACS-R parents/caregivers and (4) Autistic adults. Two members of the research team were present during each focus group, one acting as moderator and the other as notetaker. The research team developed a moderator guide for each focus group (see Supplementary materials) based on the individual aims of each focus group, with key and additional questions, however, further questions were asked based on the group discussion. Participants were also given a description of the proposed MCH workforce training to be undertaken and were able to ask clarifying questions to aid in their response to the focus group questions. The sessions occurred around the Australian summer and school holidays (approximately 6 weeks), with all focus groups audio recorded.

##### 2.2.2.1. Metropolitan and rural/regional parents/caregivers focus groups

The aim of the metropolitan and rural/regional parents/caregivers focus groups were to identify participants’ experiences with MCH nurses, referral processes, and both access and barriers to local health services. Their thoughts on the state-wide rollout of the SACS-R tool and recommendations for the MoSAIC training were also sought. Participants completed a participant information and consent form and brief family demographic survey prior to their focus group.

The metropolitan community focus group was held in-person in the Melbourne suburb of Bundoora, with two parents/caregivers from the local area participating. The rural/regional community focus group was held in-person in the regional city of Bendigo and six parents/caregivers from the region participated in this focus group. Both focus groups were approximately 60 min long. The selection criteria for this sample were that the participant lived in Victoria, was over 18 years of age, had a child aged 30 months or younger who attended the MCH service, and had no previous experience with the SACS-R.

##### 2.2.2.2. SACS-R parents/caregivers focus group

The aim of the SACS-R parents/caregivers focus group was to establish participants’ experiences with the SACS-R and autism screening in general, referral, diagnosis, and services, in addition to their overall experiences with the MCH service. These parents/caregivers had previously been involved in research on the use of the SACS-R tool (22). Participants completed a participant information and consent form and brief family demographic survey prior to their focus group.

The SACS-R sample focus group was held in-person in Bundoora and comprised of five parents/caregivers (all from the metropolitan area). The SACS-R parent/caregiver focus group was approximately 90 min long. The selection criteria for this sample were that the participant lived in Victoria, was over 18 years of age, and had previously been involved in the SACS-R research project at the Olga Tennison Autism Research Centre (OTARC).

##### 2.2.2.3. Autistic adults focus groups

The aim of the Autistic adult focus group was to gain insight on experiences with diagnosis, referral, and support pathways from a lived experience perspective. These participants were also asked for their views on and recommendations for the PDP. Participants in this focus group completed a participant information and consent form before the session commenced.

The Autistic adults focus group was held online with five participants from around Australia and lasted approximately 80 min. The selection criteria for this sample were that participants lived in Australia, were aged at least 18 years, and had been diagnosed with an autism spectrum disorder. Prospective participants were Autistic adults who had previously engaged with the study team in an advisory role and were invited to take part via email correspondence by the senior author (JB).

#### 2.2.3. Training needs survey

A training needs survey was conducted with the MCH workforce, which aimed to establish participants’ autism and developmental surveillance knowledge and experience; training requirements in autism and early social communication development; and preferences for training content and methods. Three pre-existing questionnaires were incorporated in this survey. Two domains (“Beliefs about capabilities” and “Positive emotions”) from the Determinants of Implementation Behavior Questionnaire (29) were adapted to capture factors that can influence behavior when new healthcare practices are implemented. The Hennessy Hicks Training Needs Analysis Questionnaire (30) was modified to suit the needs of MCH nurses and their practice. Participants were asked to rate how important 33 MCH workplace activities (e.g., getting on with your colleagues; inputting accurate child development data into written or computerized records; assessing a child’s clinical needs), were to successful performance at work as well as rating their current performance in these areas. An autism knowledge questionnaire, adapted from Shrestha et al. (31) and Waddington et al. (32), was also included. It comprised 39 items relating to knowledge of early childhood social communication development in both Autistic and non-Autistic children. The internal reliability analysis was acceptable (*α* =0.76).

To be eligible to participate, participants had to be over 18 years of age and currently employed in the Victorian MCH workforce. The survey link was sent to the email addresses of members of the MCH workforce supplied by the Victorian DET on 10 December 2018, which included MCH nurses, students, telephone counsellors, managers, coordinators, team leaders, and other related roles (*n* = 1,428). The survey opened on 11 December 2018 and closed on 30 January 2019, with reminders emailed every 2 weeks over the survey duration. The training needs survey had 350 respondents (24.8% response rate). After applying exclusion criteria, the final sample was 302 participants. We excluded 48 participants: two student nurses and 16 participants who did not conduct well-baby checks in their roles; 28 participants who only completed approximately one-third of the survey; and two participants who completed the survey twice, with their second response deleted.

### 2.3. Training design

The broad content of the MoSAIC training was based on that used for the SACS-R study (22), with changes and additions to the content based on the information gathered during the TNA. The curriculum addressed the following areas of training need:

- Autism knowledge
- Monitoring infants and toddlers for autism
- MCH nurse preference for autism surveillance
- Initiating conversations with families regarding autism surveillance and children’s likelihood for autism
- Communication and raising concerns with families
- Effectiveness of the referral system
- Support and resources provided to families
- Self-monitoring and reflection on autism practices to improve skills and care

During the training design process, the study team ensured that the insights gained from the lived experiences of Autistic adults and parents/caregivers were prominent in the final PDP. Based on feedback obtained from the Autistic adults, a conscious decision was made to ensure that the MoSAIC training instructed nurses to talk about autism explicitly when discussing the SACS-R assessment and a child’s likelihood of autism, rather than avoiding the word autism, in contrast with the training for the initial SACS study (20), with the intention that this would aid in reducing stigma.

A blended learning approach was used in the development of the MoSAIC training to maximize learning outcomes and to accommodate different learning styles. To facilitate this, a combination on online and in-person methods were used (Figure 1). The final MoSAIC training consisted of three components: (1) online pre-workshop modules; (2) face-to-face training workshops; and (3) online post-workshop modules.

#### 2.3.1. Resources

Based on the TNA, several learning/training resources were created to provide further support to MCH nurses and families. They are explained below.

##### 2.3.1.1. SACS-R checklists

In addition to the SACS-R checklists (for the 12-, 18-, and 24-month MCH checks) being included in the MCH electronic data system (i.e., the standard system used to capture all child health information like height, weight, etc.), training participants were also provided these checklists in hard copy.

##### 2.3.1.2. Referral pathway poster

**Figure 2:**
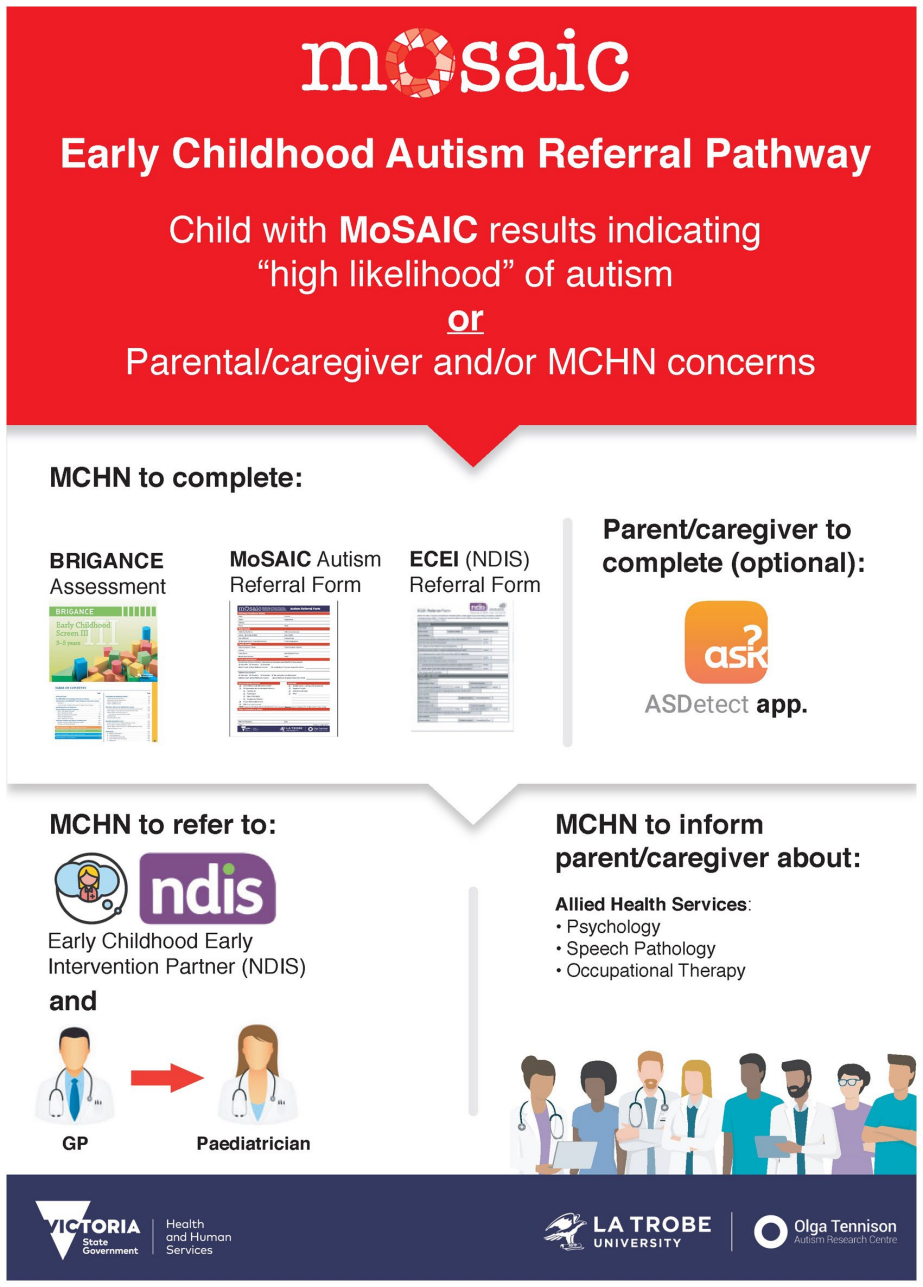
Poster of the recommended referral pathway for children identified with a high likelihood of autism.

A poster of the recommended referral pathway (Figure 2) was developed based on the feedback from the Referral Pathways Workshop. The referral pathway was intended to be followed for children identified with a high likelihood of autism, and instructed MCH nurses to refer children for diagnosis, as well as supports and services. This was designed to be displayed in MCH nurses’ rooms, to support the MCH workforce and parents/caregivers to understand how best to pursue further diagnostic assessment.

##### 2.3.1.3. SIGNS poster

**Figure 3:**
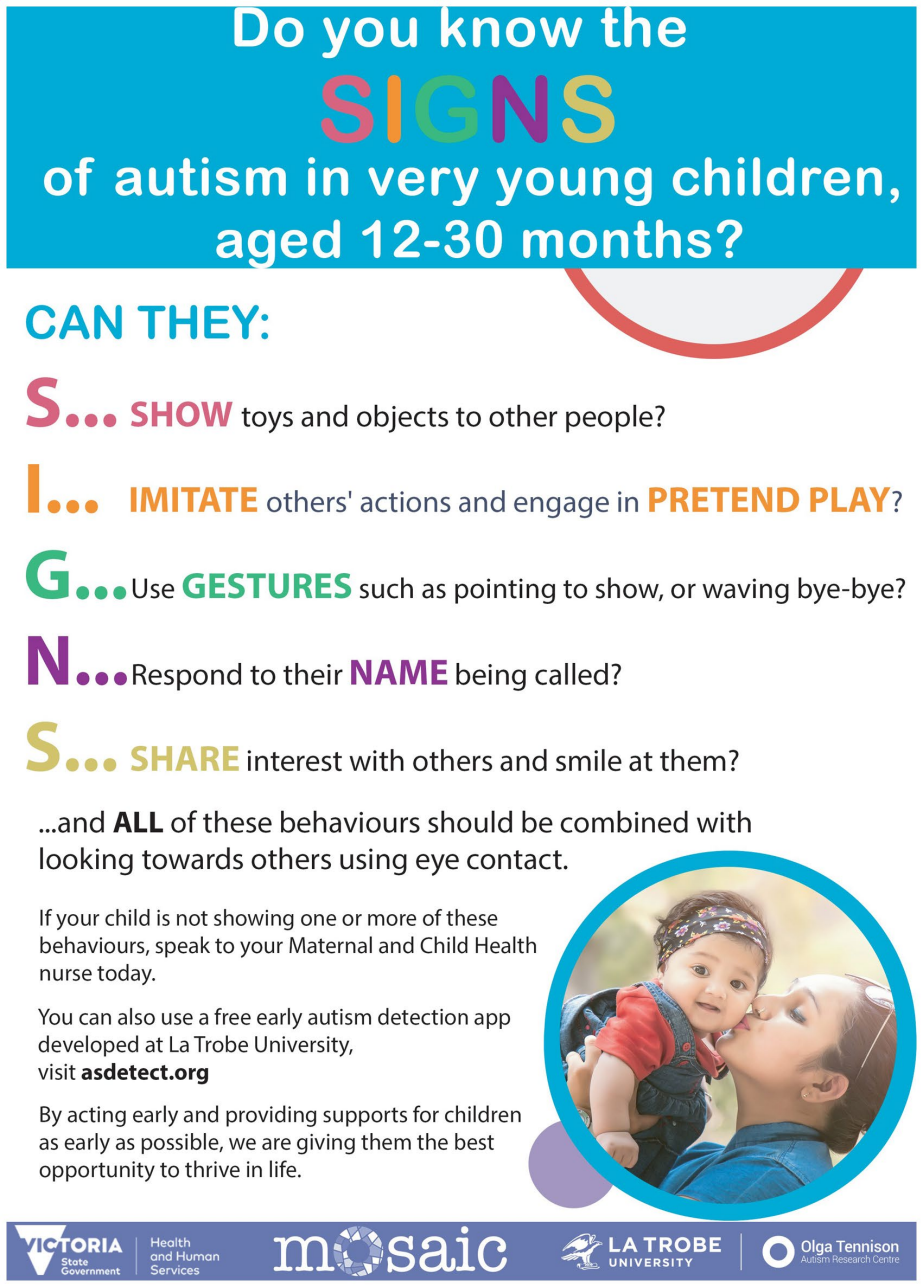
SIGNS poster.

A poster was conceptualized and developed by the senior author (JB; Figure 3), which explained five key social attention and communication behaviors in an easy-to-read format, using the acronym SIGNS (‘Show’, ‘Imitate’, ‘Gestures’, ‘Name’, ‘Share’). It also provided brief information on the ASDetect early autism detection mobile app, also developed by JB and her team (33). The SIGNS poster was intended to be placed on display in MCH centers and designed to support parent/caregiver awareness and discussion of these social attention and communication behaviors with MCH nurses.

##### 2.3.1.4. Promoting social attention interaction and communication skills booklet

Useful strategies for families to promote the development of social attention and communication skills in infants and toddlers were compiled into an eight-page printed booklet for the MCH workforce to provide to families at well-baby consultations. The booklet is available to download free of charge from the Victorian Department of Health website,^2^ with translations available in Arabic, Burmese, Chinese (simplified), Dari Farsi (Persian), Khmer (Cambodian), Punjabi, Spanish, and Vietnamese.

##### 2.3.1.5. Referral form for autism assessment

Based on suggestions received in the Referral Pathways Workshop, a referral form for autism assessment was developed. The form was designed to be completed by MCH nurses for children identified as having a high likelihood for autism, to be provided to parents to present to relevant providers, such as GPs, pediatricians, allied health services, and the National Disability Insurance Scheme (NDIS) Early Childhood Early Intervention (ECEI) staff. ECEI enables children with a developmental delay or disability under the age of 7 years to access funded supports and services without the requirement of a formal diagnosis (34), with referral to the service permitted from a variety of sources, including MCH nurses and parents/caregivers. The form comprised SACS-R results and results of other tools if applicable [e.g., ASDetect, Brigance (35)], recommended actions, and included space for child, family, and MCH nurse details. Hard copies and digital access to the Referral Form was provided to the MCH workforce.

##### 2.3.1.6. ASDetect app pamphlets

Existing pamphlets for the ASDetect (33) app were also provided as a resource for MCH nurses to give to families. The pamphlets explained that ASDetect is an app available free of charge on Apple and Google apps stores or via the website,^3^ and that it is an evidence-based parent-led early autism detection mobile app.

### 2.4. Training implementation

#### 2.4.1. Pre-workshop modules

The pre-workshop modules were developed to provide a standard level of knowledge for all MoSAIC training participants, regardless of their prior training or experience. Topics included background information about the SACS-R tool, general information about autism, and links to additional relevant resources. The modules contained a combination of podcasts, webinars, online articles, and academic papers, providing participants with different learning modes. The pre-workshop modules were hosted online on a cloud-based learning management system, allowing for self-paced learning, at a convenient time and location. Access to the pre-workshop modules commenced in March 2019, with the link to access to the pre-workshop modules sent to the email addresses of members of the MCH workforce supplied by the Victorian DET. Additional members of the MCH workforce contacted OTARC directly to register their email to access the pre-workshop modules.

#### 2.4.2. Training workshops

The face-to-face MoSAIC training workshops covered relevant areas of early social attention and communication development, and the use of the SACS-R tool. The content included an introduction to the evidence-base supporting the monitoring of social attention and communication behavior in infants and toddlers, and the differences in social attention and communication behavior in Autistic and non-Autistic children under 3 years of age. Participants were trained in the use of the SACS-R, how to integrate the SACS-R assessment into their standard 12-month, 18-month, and 24-month KAS consultations, and how to enter the SACS-R results into the MCH electronic data system. The workshop also covered strategies to discuss the administration and results of the SACS-R with parents/caregivers and a recommended referral pathway. Specific advice was provided on discussing a high likelihood result with families, approaches that were culturally sensitive, including for First Nations people (Aboriginal and Torres Strait Islanders), and what to do to support families that are not ready to act on referrals. During the workshops, participants were also provided with and instructed on how to use the resources developed as part of the PDP package (described above). The training workshops also took a multi-modal approach, using a combination of lecture, video, demonstration, and role-play. Each 5-h workshop included a 15-min and a 30-min break.

Members of the MCH workforce for whom OTARC had an email address (provided by the DET or directly from the staff member) were emailed a link to a website to enable them to register for the workshop of their choice. From 1 May to 19 June 2019, 29 training workshops were delivered across Victoria, with 20 in metropolitan Melbourne and nine in rural and regional areas. The venues were spread across the state – including locations such as Mildura (550 km from Melbourne), Wodonga (300 km from Melbourne), Hamilton (288 km from Melbourne), and Traralgon (164 km from Melbourne). Workshop locations were determined based on recommendations from DET and availability of suitable venues, with some locations hosting multiple workshops due to the centrality of these locations. A total of 1,428 members of the MCH workforce attended a face-to-face training workshop, with an additional 107 registering for a workshop who did not attend.

#### 2.4.3. Post-workshop modules

The post-workshop modules were hosted online on the same cloud-based learning management system as the pre-workshop modules. The post-workshop modules included content from the training workshops, additional material, and digital copies of the MoSAIC training resources. Face-to-face workshop attendees were provided access to the post-workshop modules after their attendance, to enhance learning and provide attendees with the opportunity to review the workshop materials. The post-workshop online modules were also designed to provide an equivalent alternative training format, for members of the MCH workforce who were unable to attend a face-to-face workshop. There were 82 participants who completed the training through the online version only (i.e., did not attend a face-to-face training workshop).

### 2.5. Training evaluation

As with the TNA, the evaluation of the MoSAIC training considered various stakeholders. There were three components of the evaluation: (1) Training Feedback Survey: a survey of the MCH workforce regarding their satisfaction with the online and face-to-face training modules; (2) Implementation Survey: a survey of the MCH workforce regarding their experiences with implementing their learnings from the PDP; and (3) Parent Evaluation Survey: a survey of parent/caregiver experiences at their child’s KAS appointment when the SACS-R assessment took place.

#### 2.5.1. Training feedback survey

This survey included the same demographic questions as the training needs survey, followed by 10 statements regarding their satisfaction with various aspects of the online and face-to-face training, and the resources provided. Participants responded to these statements using a five-point Likert scale (“strongly agree” to “strongly disagree”). Participants were asked about their knowledge and confidence in identifying the early signs of autism, as well as their confidence in implementing the training in their clinical practice. They were also able to provide any further verbatim feedback, if relevant.

To be eligible to participate in this survey, participants needed to be members of the Victorian MCH workforce, who were over 18 years of age and had completed either face-to-face or online training as part of the MoSAIC training. The survey was sent to email addresses provided by members of the MCH workforce when they registered for the training (*n* = 1,510). The emails were sent approximately one week after their face-to-face training session (i.e., in May and June 2019) or, for those who had completed only the online training, in August 2019. A reminder email was sent one week later. Participants were required to complete a participant information and consent form before access to the survey was granted. The participant information and consent form also provided consent for the implementation survey. The training satisfaction survey was completed by 344 members of the MCH workforce, a 22.8% response rate.

#### 2.5.2. Implementation survey

The implementation survey was used to identify participants’ level of knowledge and confidence in implementing the SACS-R tool in their clinical practice, as well as other learnings from the PDP. Participants were also asked questions relating to their experiences implementing the SACS-R tool as a part of their routine KAS consultations. Some of the questions in the implementation survey were based on questions that were included in the training needs survey, including the Practice Behavior Questionnaire (29).

Participants who had completed the training feedback survey were invited to complete the implementation survey. An email invitation to participate in this survey was sent 4 to 6 weeks after training workshop completion, to allow time for participants to implement their learnings into their routine practice, with a reminder email sent one week later. A total of 113 participants completed the implementation survey, representing 7.48% of training attendees and 34.35% of those who had completed the training feedback survey.

#### 2.5.3. Parent/caregiver evaluation survey

In terms of recruitment, members of the MCH workforce who had completed the in-person or online training were provided with business cards with brief information about the survey and a link and QR code to the participant information and consent form. They invited eligible parents/caregivers who had attended a 12-, 18-, or 24-month KAS appointment, where a SACS-R assessment was completed, to participate.

This survey contained questions regarding child and family demographic information, as well as parent/caregiver experiences of MCH visits, administration of the SACS-R tool, and referral(s) to other medical or allied health professionals, where relevant.

To be eligible to complete the survey, parents/caregivers needed to live in Victoria, be at least 18 years of age, have a child aged 30 months or younger, and have attended the MCH service between May and August 2019.

As the initial recruitment of participants for the parent evaluation survey was lower than expected, a more targeted approach was taken with an SMS message broadcast. The survey was sent to an additional 1,000 parents/caregivers who met the selection criteria (i.e., their child 30 months of age or younger had attended an appointment with and MCH nurse who had completed the MoSAIC training between May and August 2019). Those who completed the parent evaluation survey were eligible to go into a draw to win one of 10 AUD $50 Coles Myer gift vouchers. A total of 48 participants completed a valid response to the parent/caregiver evaluation survey.

### 2.6. Statistical analyses

#### 2.6.1. Qualitative analyses

Transcripts from the referral pathways workshop and all focus groups were analyzed thematically, allowing the identification of key themes for each respective topic. Data for each of the focus groups were manually coded and organized separately by the team members present during the session, thus enabling comparisons between the groups where relevant. Discussion was used to reach consensus on themes.

#### 2.6.2. Quantitative analyses

Statistical analyses were conducted using SPSS Statistics for Windows, version 29.0 (36). Descriptives and frequencies were calculated for all quantitative data, with chi-square analysis used to compare scores between groups (all tests were 2-sided *α* = 0.05). For the Hennessy-Hicks Training Needs Analysis Questionnaire, the ‘improvement’ score was calculated by determining the difference between the mean ‘performance’ score and the mean ‘importance’ score.

## 3. Results

### 3.1. Training needs analysis

#### 3.1.1. Referral pathways workshop

All participants were female, which is not surprising given the very small number of male members of the MCH workforce (37, 38) Twenty participants (46.5%) reported working in a metropolitan area, 19 (44.2%) in a rural/regional area, and four (9.3%) did not specify.

**Figure 4:**
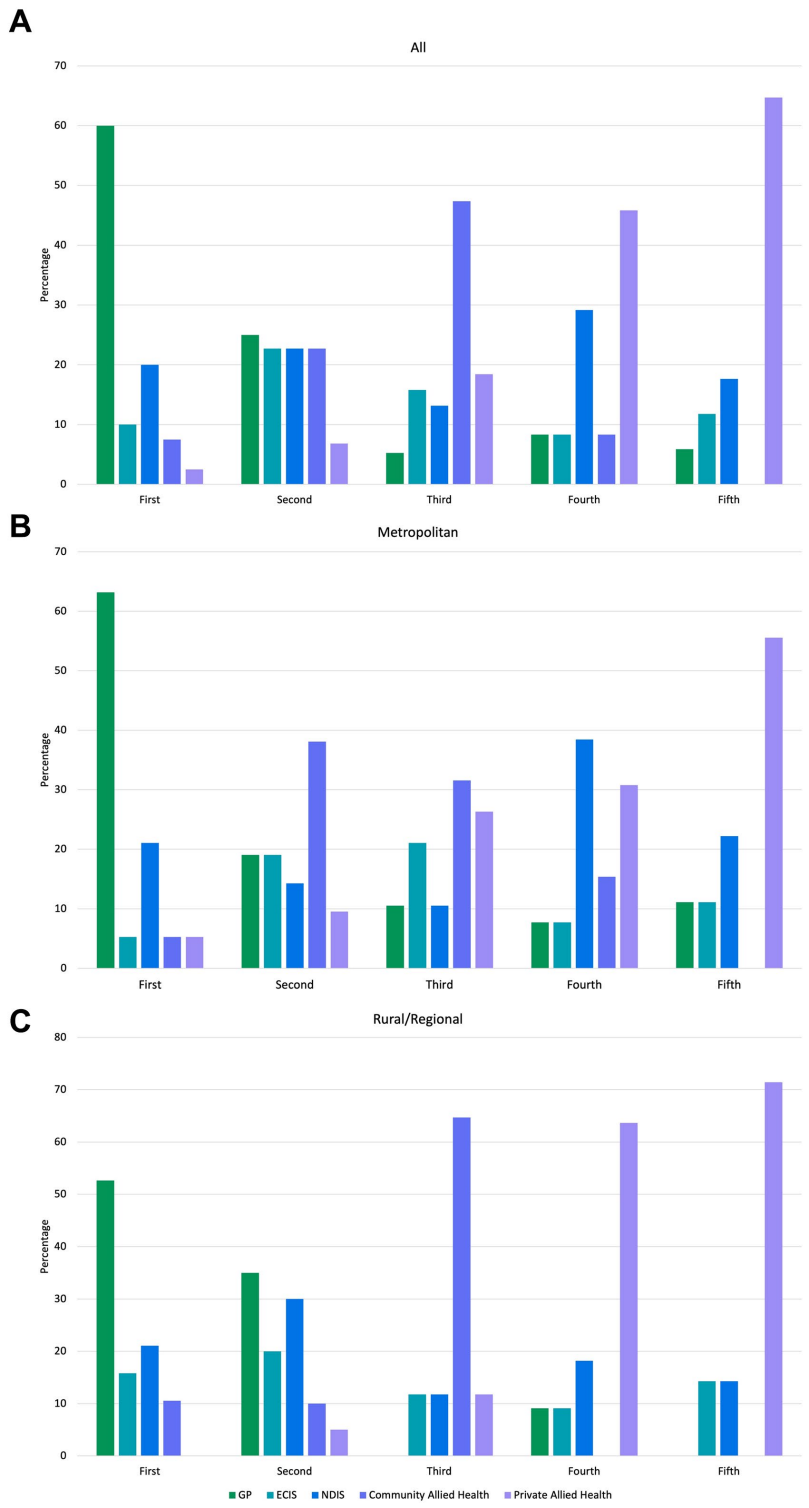
Diagram of order of referral service from the referral pathways workshop.

The first referral made by most participants was to a GP (60.0%), followed by the ECEI program through the NDIS (20.0%) as the second referral service (Figure 4). Community Allied Health (state-funded community allied health services) was the service most referred to third (47.4%), with private Allied Health services the most common as fourth and fifth referrals (45.8 and 64.7%, respectively).

When analyzed by geographic location, GPs were the most commonly ranked first service to which nurses referred a child with a high likelihood of autism, by both metropolitan nurses (*n* = 12, 62.3%) and rural/regional nurses (*n* = 10, 52.6%). GPs were also the highest service ranked second service children were referred to, by rural/regional nurses (*n* = 7, 35%), with the NDIS ECEI service (*n* = 6, 30%) the next highest ranked as the second service. For nurses in metropolitan areas, Community Allied Health was the service most nominated as their second referral preference (*n* = 8, 38.1%). Only 21.1% (*n =* 4) of metropolitan nurses ranked the NDIS ECEI as the first place they would refer to, with the majority (*n =* 5, 38.5%), ranking this service provider as their fourth preference. A large percentage of rural/regional nurses (*n =* 11, 64.7%) ranked community allied health as their third preference for referral, and both community-and private-based allied health were most commonly ranked third by participants in the metropolitan area (*n =* 6, 31.6%; *n =* 5, 26.3%, respectively).

Barriers to accessing referral pathways were identified. Parental/caregiver reluctance to accept that their child had a high likelihood of autism, and therefore parental/caregiver reticence to start the process of accessing diagnosis and supports, were key barriers identified in the metropolitan group. Participants reported difficulty in deciding between making an appropriate referral (i.e., for diagnosis) that might not be received well by families or referring families to a service that they were more likely to accept (e.g., speech pathology or occupational therapy) as these services are often viewed as general services and not only for Autistic children. State-funded primary health services rejecting referrals in cases where a child had complex needs was another key issue identified. When requests from MCH nurses to GPs (to refer a child to a pediatrician for further investigation) were not accepted by GPs, these instances were also seen as obstructive, especially as nurses cannot refer directly to pediatricians in Victoria.

Those in the rural/regional break-out session noted a high level of difficulty when referring families to ECEI services, citing inconsistency in referral requirements and paperwork between ECEI service providers, and a high level of parent/caregiver burden when trying to access ECEI services. Referrals by MCH nurses to ECEI services were also not accepted in some areas. Both the metropolitan and rural/regional groups identified that long waiting lists for services such as ECEI (1–6 months in metropolitan areas and 3–8 months in rural/regional areas at the time the study was conducted) and community allied health also created difficulties for families, with costs of private allied health services prohibitive to many.

#### 3.1.2. Focus groups

##### 3.1.2.1. Metropolitan and rural/regional parent/caregiver focus groups

All participants in the metropolitan and rural/regional parent/caregiver focus groups were female and the primary caregiver, with a mean age of 32.2 years (SD: 5.78; range: 26.1 to 40.5 years). No participants reported being Aboriginal and/or Torres Strait Islander; 83.3% (*n =* 5) were Australian born and reported Australian ethnicity. English was spoken at home by all participants, with 16.7% (*n =* 1) also speaking a language other than English at home. Most participants (*n =* 5, 83.3%) reported that they were married or in a *de facto* relationship.

When asked about their general experiences with MCH nurses and well-baby checks, participants reported a mix of exceptional and less positive experiences. Several participants discussed situations where they felt their MCH nurse had gone beyond expectation. Positive aspects included MCH nurses building rapport with them, taking extra time when necessary, and being proactive in providing support to the child and family when needed. Participants relayed that during such experiences, they felt they could trust their MCH nurse and that the nurse truly listened to them. Such experiences were not limited to participants’ regular MCH nurses, but to MCH nurses in general, even when only seen for a single appointment, with no previous history between nurse and family. When describing less positive experiences, participants noted occurrences where MCH nurses did not listen to them or try to develop a relationship with them, or were overly vigilant, which caused stress to the family. Participants also reported inconsistency in MCH staffing to be an issue.

Experiences with general referrals were varied, and metropolitan participants largely found services easy to access. One participant described issues with accessing support for herself and noted that her MCH nurse assisted her with resolving this. In contrast, those in the rural/regional areas described long waiting lists in the public health system. They noted that while urgent issues were dealt with quickly, small problems were often not.

The idea of introducing the SACS-R into standard MCH well-baby checks for all Victorian children received a positive response. Participants believed that most other parents/caregivers would feel similarly, even in instances where finding out that a child has a high likelihood of autism might be upsetting. They agreed that early identification would help families to have a better understanding of their child’s needs and enable them to access necessary services. Participants supported the idea that normalizing the administration of the SACS-R would reduce stigma about autism and reduce stress within families, and that the SACS-R procedure should be presented to families in the same way as any other part of a well-baby assessment (i.e., weight, height, and hearing). Participants thought that a brief explanation of the procedure should be supplied but no more so than other standard checks. It was also felt that standard inclusion of the SACS-R in well-baby checks would help to normalize autism within the community.

Participants also gave several recommendations for inclusion in the training program. To benefit families, they suggested providing education on (1) how to communicate a high likelihood result, (2) how to support families after the result, and (3) how to offer hope for their child’s future. To empower parents/caregivers to be able to monitor their child’s behavior at home, several participants suggested that MCH nurses should be trained in educating parents/caregivers in typical and atypical social attention and communication.

##### 3.1.2.2. SACS-R parents/caregivers focus group

Participants in the SACS-R parents/caregivers focus group were all female, the child’s primary caregiver, and had a mean age of 37.3 years (SD: 4.66; range: 32.2 to 42.2). Sixty percent (*n* = 3) were born in Australia, 80% (*n* = 4) reported non-Australian ethnicity, and none reported Aboriginal and/or Torres Strait Islander origins. All participants spoke English at home; 20% (*n* = 1) also spoke a language other than English at home. Most participants (*n* = 4, 80%) were married or in a *de facto* relationship.

When reporting on their general experiences with MCH nurses and well-baby checks, participants reported a mix of exceptional and less positive experiences. One participant reported that she was so pleased with her MCH nurse that she continued to see her despite moving over 45 min away from the Centre.

While some participants described not realizing that their child had any behavioral differences prior to their MCH nurse completing the SACS-R assessment, others said it affirmed their concerns about their child’s development. Generally, participants spoke favorably of the SACS-R identification process and of having their child identified as at high likelihood. They felt the process was easy and informative, and crucial in getting supports for their child and most believed their MCH nurse was proactive in encouraging them to seek supports and services for their child. Most reported challenges in navigating access to services and long waiting lists, with little follow up or support. Many participants were concerned about what they should be doing to help their child while waiting to access services.

The introduction of the SACS-R into standard MCH well-baby checks for all Victorian children received a positive response from the SACS-R parents/caregivers focus group sample as did the proposed training program for the MCH workforce. The SACS-R parents/caregivers also provided specific suggestions for inclusion in the MoSAIC training, including training MCH nurses to: (1) inform parents/caregivers correctly and clearly about a high likelihood of autism, (2) convey next steps clearly, and to instill how important it is to start these steps as soon as possible, and (3) offer advice on what families can do while waiting to access services. It was also suggested that MCH nurses’ follow-up with parents/caregivers after communicating a high likelihood result, to check in with families, and to establish whether they have started the diagnostic process, or need additional information. Creating autism information resources, in multiple formats and languages was suggested to make information more accessible for a wide range of abilities and cultures, including grandparents, and extended family members.

##### 3.1.2.3. Autistic adults focus group

Demographic information on the Autistic adult focus group was not collected in order to maintain confidentiality. The group agreed that early identification and diagnosis of Autistic children was beneficial. Some participants noted that being undiagnosed or misdiagnosed well into adulthood caused them to struggle and experience negative effects, including poor mental health. They expressed hope that the MoSAIC training would help to prevent children from experiencing the disadvantages of being an undiagnosed Autistic person. They also hoped the MoSAIC training would in turn help to educate both professionals and the general public about autism and promote increased support and better understanding. Concerns were expressed about the challenges that MCH nurses may experience in screening for autism. This included difficulties in successfully identifying Autistic children who have a non-conventional or less clear presentation, as well as concerns around MCH nurses having limited time and capacity to implement thorough autism screenings during allotted appointments, given the myriad general assessments also conducted in a MCH consultation.

Further topics of discussion included the importance of ‘next steps’ after a high likelihood identification by an MCH nurse. Participants highlighted the importance of supports and services being made available soon after identification. Group members emphasized the value of families receiving a balanced message about autism, suggesting that families should not only be given the ‘good news’ stories, but also information on the challenges of autism. They also noted that families needed to be connected into the autism community, so that the child is aware that they are part of a community and not alone. Participants stressed that using the word ‘autism’ was not a bad thing and it should not be avoided – they preferred their correct “label” be used rather than being labeled with a negative or derogatory term. There was group consensus that the use of the term “surveillance” was problematic as it has negative connotations, with “monitoring” suggested as a favorable alternative, thus leading to the name MoSAIC for the training program.

#### 3.1.3. Training needs survey

All participants were female, which was expected due to the overwhelming female-majority in the MCH workforce [(37, 38); Table 1]. There were 206 (68.2%) participants working in the metropolitan area and 82 (27.2%) in rural/regional areas, with work locations of 14 (4.6%) participants undisclosed. Almost two-thirds (*n* = 198, 65.6%) of participants were aged over 50 years and close to half (*n* = 150, 49.6%) reported having practiced as an MCH nurse for over 10 years. The most reported current professional title was ‘MCH nurse’ (*n* = 221, 73.2%), followed by ‘Enhanced MCH nurse’ (*n* = 39, 12.9%). Most participants reported ‘postgraduate diploma’ as their highest completed level of education (*n* = 204, 67.5%). A substantial number of nurses reported working ‘more than 30 h’ a week (*n* = 117, 38.7%), though these work hours were more commonly reported by metropolitan (*n* = 89; 43.2%) than rural/regional participants (*n* = 24, 29.3%).

**Table 1:** Training needs survey and training feedback survey participant demographics.

	Training needs analysis (*N* = 302)		Training feedback survey (*N* = 344)		*p* value
	*n*	%	*n*	%	
Gender					
Female	302	100.0	342	99.4	0.537
Male	0	0.0			
Indeterminate/Intersex/Unspecified	0	0.0	2	0.6	
Age (years)					
18–25	0	0.0	2	0.6	0.064
26–30	3	1.0	14	4.1	
31–35	15	5.0	32	9.3	
36–40	20	6.6	23	6.7	
41–45	26	8.6	31	9.0	
46–50	39	12.9	42	12.2	
51–55	66	21.9	53	15.4	
56–60	78	25.8	80	23.3	
61 or over	54	17.9	65	18.9	
Prefer not to say	1	0.3	2	0.6	
Highest level of education completed					
Certificate or hospital qualification	4	1.3	4	1.2	0.841
Diploma	11	3.6	12	3.5	
Undergraduate (Bachelor’s) Degree/s	13	4.3	22	6.4	
Postgraduate Diploma/s	204	67.5	215	62.5	
Master’s Degree/s	68	22.5	89	25.9	
Doctoral Degree/s	1	0.3	1	0.3	
Other	1	0.3	1	0.3	
Years of practice as an MCH nurse					
Less than 3	34	11.3	66	19.2	0.033
3–5	44	14.6	33	9.6	
6–10	74	24.5	87	25.3	
11–15	43	14.2	43	12.5	
More than 15	107	35.4	115	33.4	
Current professional title^a^					
MCH center nurse	221	73.2	214	62.2	
MCH nurse reliever	30	9.9	34	9.9	
MCH Site Coordinator/Team leader/Manager	17	5.6	49	14.2	
Enhanced Home Visiting MCH nurse	39	12.9	42	12.2	
ACCO MCH nurse	4	1.3	5	1.5	
MCH Line Telephone Councilor	11	3.6	6	1.7	
EPC nurse	7	2.3	6	1.7	
MCH nurse student			30	8.7	
Other	6	2.0	8	2.3	
Average hours worked per week					
Less than 7	3	1.0	23	6.7	0.004
7–15	29	9.6	38	11.0	
16–25	98	32.5	107	31.1	
26–30	55	18.2	47	13.7	
More than 30	117	38.7	129	37.5	
Area workplace is located^b^					
Metropolitan area	206	71.5	239	70.7	0.822
Rural/regional area	82	28.5	99	29.3	

^a^Participants were able to select more than one professional title if they worked in multiple roles. Therefore, differences between the groups were not able to be tested. ^b^Those with multiple workplaces were asked to answer regarding their main role. ACCO, Aboriginal Community Controlled Organization; EPC, Early Parenting Centre; MCH, Maternal and Child Health.

**Figure 5:**
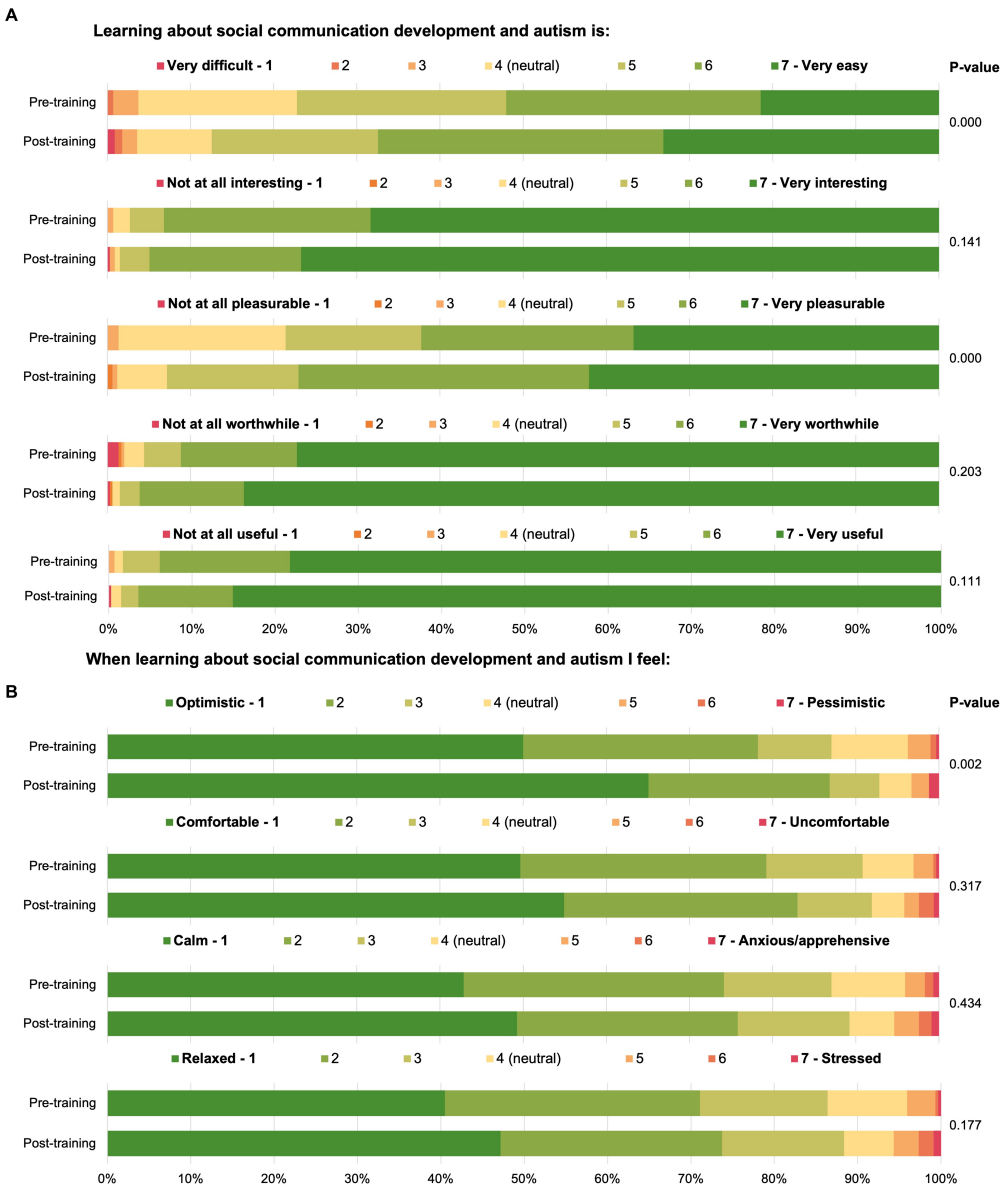
Maternal and child health workforce members’ feelings regarding learning about autism and social communication.

Almost all respondents felt learning about autism and social communication development was useful (*n* = 289, 98.2%), worthwhile (*n* = 281, 95.5%), and interesting (*n* = 286, 97.3%; Figure 5). However, in total over half had either never completed any formal training on autism (*n* = 28, 9.3%) or had not done so in the last 2 years (*n* = 140, 46.4%). Almost all participants ‘agreed’ or ‘strongly agreed’ (*n* = 300, 99.7%) with the statement “MCH nurses have an important role to play in the identification of autism,” though only 37.6% (*n* = 113) ‘agreed’ or ‘strongly agreed’ that they had adequate tools and resources within their workplace to support children who are Autistic or who had a high likelihood for autism. When asked about the availability of adequate supports and services for families of children who are Autistic or identified as having a high likelihood for autism, agreement decreased to less than a quarter (*n* = 74, 24.6%). Similarly, less than one-third of participants (*n* = 96, 31.9%) felt that the recommended referral pathway in their local area is effective.

Inconsistency in responses to items about autism knowledge and social development indicated irregularity in current knowledge within these areas. No participants indicated that vaccines are a possible cause of autism, and almost all agreed with statements such as “a 12–24 month old child responds when someone calls his/her name” (*n* = 295, 97.7%), “an 18-month old child speaks 5–10 clear words” (*n* = 293, 97.0%), and “generally, Autistic children prefer sameness in their routine such as eating the same food or taking the same route to go to school” (*n* = 291, 96.4%). However, almost one in ten (*n* = 22, 7.3%) respondents agreed that children aged 12–24 months are not interested in other children their age, with a similar number believing that Autistic children do not show repetitive movements (*n* = 31, 10.3%) or repetitive patterns of speech (*n* = 24, 7.9%). Participants were also asked about their confidence in monitoring the signs of autism in young children. A greater proportion of nurses reported feeling confident in doing so with 24-month-old children (*n* = 265, 90.1%), compared to 12-and 18-month-olds (respectively *n* = 194, 66.0%; *n* = 243, 82.7%).

Participants were asked about the barriers they experienced in supporting children with a high likelihood for autism and their families. Long waiting lists for appropriate services in their area (*n* = 248, 83.2%), the belief that suggesting an autism referral would upset parents/caregivers (*n* = 139, 46.7%), and GPs not providing appropriate referrals to a pediatrician (*n* = 123, 41.3%) had the highest level of agreement. Close to one in five (*n* = 53, 17.8%) participants agreed that they would find suggesting an autism referral uncomfortable.

When asked what training or resources would be most useful when learning about autism, the most selected response options were “a toolkit for screening and diagnosis of autism” (*n* = 249, 82.5%) and “a checklist/flowchart of community resources for parents/caregivers of Autistic children” (*n* = 223, 73.8%). Around two thirds of participants also selected training on effective strategies for communicating with parents/caregivers (*n* = 198, 65.6%) and children with a high likelihood for autism (*n* = 187, 61.9%).

The Hennessy-Hicks Training Needs Analysis Questionnaire was used to determine knowledge areas that had the greatest training need. Eight of the top 10 needs were common to both metropolitan and rural/regional participants (Table 2). The areas of greatest training need were: instructing or training students or junior staff in the early detection of autism (all: mean difference = 1.58; metropolitan: mean difference = 1.39; rural/regional: mean difference = 1.91); and appraising their own performance in recognizing the early signs of autism (all: mean difference = 1.49; metropolitan: mean difference = 1.41; rural/regional: mean difference = 1.73). Other areas identified as training needs were assessing a child’s likelihood for autism related to social communication delays (all: mean difference = 1.23; metropolitan: mean difference = 1.07; rural/regional: mean difference = 1.55); making decisions about the specific needs of children at high likelihood for autism (all: mean difference = 1.16; metropolitan: mean difference = 1.05; rural/regional: mean difference = 1.46); and several items relating to communicating with parents and caregivers, such as explaining developmental surveillance, information, and assessment methods to families. Nurses also wanted to increase their capability in understanding the quality of research on the early detection of autism (all: mean difference = 1.73; metropolitan: mean difference = 1.73; rural/regional: mean difference = 1.84).

**Table 2:** Areas of greatest training need - MCH workforce.

All participants	Mean difference
Critically evaluating the quality of research on the early detection of autism in children under 31 months	1.73
Instructing or training students/junior staff in the early detection of autism	1.58
Appraising your own performance in recognizing the early signs of autism	1.49
Assessing a child’s “likelihood for autism” associated with social communication delays	1.23
Identifying areas of clinical practice that should be systematically investigated	1.16
Making decisions about the specific needs of children at “high likelihood of autism”	1.16
Finding information that can inform your clinical work	1.10
Knowing how to use equipment and information to explain developmental surveillance to parents/caregivers	1.06
Advising parents/caregivers on developmental surveillance methods	1.03
Introducing new ideas into your own maternal and child health work	1.02
Providing feedback to colleagues working in maternal and child health	0.98
Metropolitan participants	
Critically evaluating the quality of research on the early detection of autism in children under 31 months	1.72
Appraising your own performance in recognizing the early signs of autism	1.41
Instructing or training students/junior staff in the early detection of autism	1.39
Identifying areas of clinical practice that should be systematically investigated	1.11
Finding information that can inform your clinical work	1.09
Assessing a child’s “likelihood for autism” associated with social communication delays	1.07
Making decisions about the specific needs of children at “high likelihood of autism”	1.05
Advising parents/caregivers on developmental surveillance methods	0.98
Introducing new ideas into your own maternal and child health work	0.97
Knowing how to use equipment and information to explain developmental surveillance to parents/caregivers	0.96
Providing feedback to colleagues working in maternal and child health	0.89
Rural/regional participants	
Instructing or training students/junior staff in the early detection of autism	1.91
Critically evaluating the quality of research on the early detection of autism in children under 31 months	1.84
Appraising your own performance in recognizing the early signs of autism	1.73
Assessing a child’s “likelihood for autism” associated with social communication delays	1.55
Making decisions about the specific needs of children at “high likelihood of autism”	1.46
Identifying areas of clinical practice that should be systematically investigated	1.32
Finding information that can inform your clinical work	1.30
Knowing how to use equipment and information to explain developmental surveillance to parents/caregivers	1.27
Introducing new ideas into your own maternal and child health work	1.25
Assessing parent/caregiver satisfaction with maternal and child health checks	1.23
Planning clients’ referral for further investigations or treatment	1.21

### 3.2. Training implementation

#### 3.2.1. Pre-workshop modules

Due to the configuration of the pre-and post-workshop modules in the same system, it was not possible to determine the number of participants who accessed the pre-workshop modules; however, access to the pre-workshop modules was a compulsory part of the PDP.

#### 3.2.2. Training workshops

There were 1,428 members of the MCH workforce who completed an in-person training workshop. Twenty of the workshops (1,038 attendees, 72.7%) were held in metropolitan Melbourne, with a further 9 workshops (390 attendees, 27.3%) held in rural/regional Victoria.

#### 3.2.3. Post-workshop modules

As specified above, it was not possible to determine the number of participants who accessed the post-workshop modules. Overall, a total of 1,528 members of the MCH workforce accessed the pre-and/or post-workshop modules at least once. This includes 82 (5.43% of all trainees) trainees who only accessed the training via the post-workshop modules.

### 3.3. Training evaluation

#### 3.3.1. Training feedback survey

Most participants were female (*n* = 342, 99.4%), consistent with the gender breakdown of the MCH workforce (37, 38). The majority (*n* = 239, 69.5%) worked in the metropolitan area, with 28.8% (*n* = 99) working in a rural or regional area, and 1.7% (*n* = 6) noting that area was not applicable for them (e.g., MCH telephone line nurses). Most (*n* = 98, 57.6%) participants were aged over 50 years, with almost a quarter (*n* = 80, 23.3%,) in the ‘56–60 years of age’ bracket. Close to two-thirds (*n* = 215, 62.5%,) nominated a postgraduate diploma as their highest level of completed education and 45.9% (*n* = 158) had practiced as a MCH nurse for over 10 years. The most commonly selected role was MCH center nurse (*n* = 214, 62.2%,) followed by MCH coordinator/team leader/manager (*n* = 49, 14.2%). ‘More than 30 h’ was the most commonly selected work hours per week (*n* = 129, 37.5%). The demographics of the training feedback survey participants were similar to those of the training needs survey participants, with only two significant differences found between the groups. For years of practice as an MCH nurse, there was a higher proportion of participants in the training feedback survey having less than three years’ experience [*X*^2^ (4, *N* = 646) = 10.46, *p* = 0.033]; for average hours worked per week, a greater proportion of respondents to the training feedback survey worked less than 7 h per week [*X*^2^ (4, *N* = 646) = 15.54, *p* = 0.004]. Both of these can be explained by the inclusion of student MCH nurses in the training feedback survey.

When asked which of the training components they had completed, 85.2% (*n* = 293) had completed the pre-workshop modules, 97.4% (*n* = 335) had attended a training workshop, and 18.6% (*n* = 64) had completed the post-workshop modules. The lower number of participants who had completed the post-workshop modules at the time of completing the survey is not unexpected given the survey was sent to participants 1–2 weeks after they had attended a training workshop, and participants may not have accessed it yet.

Almost all (*n* = 272, 93.8%) participants agreed that the pre-workshop module material was clear and well presented, and that the pre-workshop modules met their expectations (*n* = 255, 87.9%). Similarly, most participants agreed that the module increased their knowledge about autism (*n* = 237, 81.7%) and that they had gained confidence in using the SACS-R tool after completing the pre-workshop module (*n* = 264, 91.3%). When asked if they would recommend the pre-workshop modules to others, 87.9% (*n* = 255) agreed.

**Figure 6:**
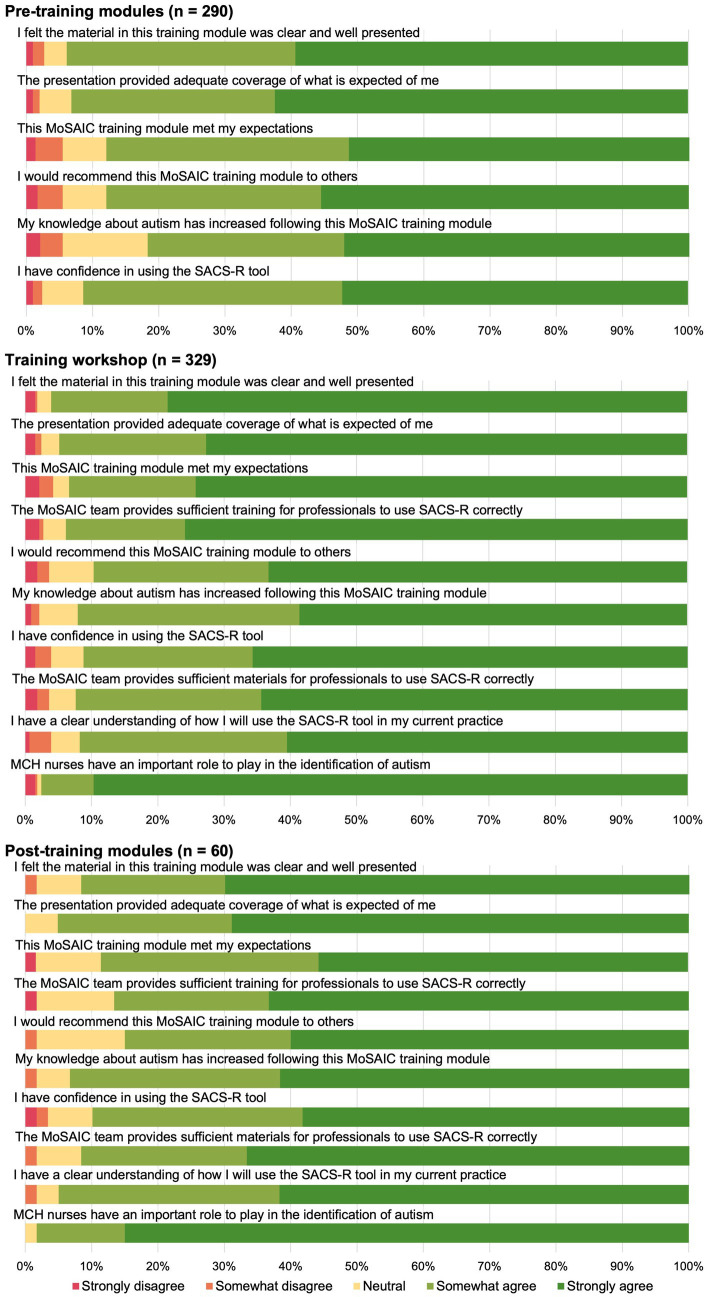
Training feedback survey for mosaic pre-training modules, training workshops, and post-training modules.

Feedback regarding the training workshops was similarly positive (Figure 6). Most participants agreed that the training workshop was clear and well presented (*n* = 316, 96.0%), met their expectations (*n* = 307, 93.3%), and that they would recommend the training workshop to others (*n* = 295, 89.7%). Most participants agreed that the training workshop provided sufficient training (*n* = 308, 93.9%) and sufficient materials for professionals to use SACS-R correctly (*n* = 304, 92.4%). Almost all participants agreed that their knowledge about autism had increased after completing the training workshop (*n* = 302, 92.1%) and that they have confidence in using the SACS-R tool (*n* = 300, 91.2%).

Participants agreed that the post-workshop modules were clear and well presented (*n* = 55, 91.7%), met their expectations (*n* = 54, 88.5%), and had increased their knowledge about autism (*n* = 56, 93.3%). Most also agreed that the post-workshop modules provided sufficient training (*n* = 52, 86.7%) and sufficient materials for professionals to use SACS-R correctly (*n* = 55, 91.7%), and that they would recommend the post-workshop module to others (*n* = 51, 85.0%). Learning about social attention and communication development in autism was reported as useful, worthwhile, and interesting (all: *n* = 330, 98.5%), and pleasurable (*n* = 311, 92.8%; Figure 5). Although ratings in these areas had improved from responses to the survey prior to training, the differences were not significant. Significant increases were found for feeling that learning about social attention and communication development in autism was pleasurable [pre-training: *n* = 231, 78.6%; post-training: *n* = 311, 92.8%; *X*^2^ (5, *N* = 629) = 33.20, *p* = 0.001] and easy [pre-training: *n* = 227, 77.2%; post-training: *n* = 293, 87.5%; *X*^2^ (6, *N* = 629) = 25.74, *p* = 0.001], and that when asked to learn about monitoring early social communication and autism, participants felt optimistic [pre-training: *n* = 256, 87.1%; post-training: *n* = 311, 92.8%; *X*^2^ (6, *N* = 629) = 21.42, *p* =0.02; Figure 5].

#### 3.3.2. Implementation survey

Participants’ responses to the autism knowledge questionnaire showed a statistically significant increase in the proportion of correct responses, indicating an increase in knowledge following the training. For example, in the implementation survey 97.3% (*n* = 110) selected the correct response to the statement “an 18- to 24-month-old child *does not* follow simple commands,” compared to 94.7% [*n* = 286; *X*^2^ (2, *N* = 415) = 9.32, *p* = 0.009] prior to training. For the statement “autism is just an intellectual disability,” 99.1% (*n* = 112) selected the correct response post-training, compared to 91.7% [*n* = 277; *X*^2^ (2, *N* = 415) = 7.76, *p* = 0.021] pre-training. Participants showed an increased understanding of early identification, with 94.7% (*n* = 107) of respondents post-training selecting the correct response to the statement “children with autism can be identified by the age of 24 months,” in comparison to 83.1% [*n* = 251; *X*^2^ (2, *N* = 415) = 10.10, *p* = 0.006] pre-training. For the statement “autism occurs more commonly in higher educational levels,” 72.5% (*n* = 219) selected the correct response pre-training, increasing to 88.5% [*n* = 100; *X*^2^ (2, *N* = 415) = 12.15, *p* = 0.002] post-training.

Responses to questions about nurses’ autism screening practices (identifying, communicating about, and monitoring for signs of autism) demonstrated an increase in self-efficacy from pre-to post-training. Pre-training, 77.6% (*n* = 228) of participants agreed or strongly agreed with the statement “I feel confident that I can identify children with a higher likelihood of developing an ASD,” which significantly increased to 98.2% (*n* = 111) post-training [*X*^2^ (3, *N* = 407) = 45.06, *p* = < 0.001]. When asked for their response to the statement “I feel *unsure* when talking to parents/caregivers about autism,” 19.0% (*n* = 58) agreed or strongly agreed pre-training, which decreased to 8.8% (*n* = 10) post-training, though the difference was not significant. Increases in confidence were also seen in participants’ capability to monitor for signs of autism in younger children, where non-significant increases occurred from pre- to post-training in 12-month-olds (pre-training: *n* = 194, 66.0%; post-training: *n* = 76, 78.4%), 18-month-olds (pre-training: *n* = 243, 82.7%; post-training: *n* = 88, 88.9%), and 24-month-olds (pre-training: *n* = 265, 90.1%; post-training: *n* = 90, 90.9%).

Almost all participants (*n* = 102, 91.1%) reported that they were currently using the SACS-R tool. Of those who reported not using the tool at the time of their response (*n* = 10, 8.9%), four (40%) were MCH nurse students who were not currently on clinical placement and an additional four (40%) noted that using SACS-R was not applicable in their role (e.g., MCH phone line counsellor, MCH team leader who does not conduct KAS assessments). Most participants reported that they automatically use the SACS-R in their daily work (*n* = 94, 94.0%) and that they intend to continue to use SACS-R (*n* = 96, 96.0%). Almost all participants agreed that they are confident they can use SACS-R correctly (*n* = 98, 97.0%) and that if they use SACS-R correctly that it benefits children and families (*n* = 100, 99.0%). Participants also reported a high level of confidence in speaking to parents/caregivers about SACS-R (*n* = 95, 94.1%) and most also agreed that parents/caregivers had been comfortable with SACS-R being undertaken with their child (*n* = 82, 81.2%).

Participants were also asked for feedback regarding the resources provided as a part of the PDP. Close to half (*n* = 47, 47.0%) found the referral form easy-to-use, with an additional 43.0% (*n* = 43) responding that they felt neutral. When asked if parents/caregivers had responded positively to the SIGNS poster, 57.1% (*n* = 56) agreed, while 41.8% (*n* = 41) were neutral. Similarly, when asked if parents/caregivers had responded positively to the Promoting Social Attention Interaction and Communication Skills booklet, 39.6% (*n* = 38) agreed and 59.4% (*n* = 57) were neutral.

#### 3.3.3. Parent/caregiver evaluation survey

Almost all respondents were the primary caregiver (*n* = 46, 95.8%) and the child’s mother (*n* = 46, 95.8%). Most primary caregivers had completed a university degree (*n* = 38, 79.2%), classified their occupation as ‘professional’ (*n* = 32, 68.1%), and were currently working part-time (*n* = 32, 66.7%). Three-quarters (*n* = 36, 75%) of primary caregivers were born in Australia, with the next most reported countries of birth being India (*n* = 3, 6.3%) and New Zealand (*n* = 2, 4.2%). Two-thirds (*n* = 32, 66.7%) of primary caregivers’ family background was ‘Australian’, with Indian (*n* = 4, 8.3%) and Italian (*n* = 2, 4.2%) being the next most reported ethnic backgrounds.

Over three-quarters (*n* = 37, 77.1%) of participants stated their child had a secondary caregiver. The majority (*n* = 35, 92.1%) of secondary caregivers were the child’s father. Similar to the primary caregivers, most had completed university studies (*n* = 38, 47.4%), however, a greater proportion had completed a trade certificate (*n* = 12, 25.0%) when compared with primary caregivers (*n* = 1, 2.1%). ‘Professional’ was also the most reported occupation for secondary caregivers (*n* = 15, 39.5%), though most (*n =* 35, 92.1%) were employed full-time. Australia was the most common (*n* = 30, 78.9%) birth country for secondary caregivers, with all other birth countries equally represented (Cambodia, China, Fiji, India, Malaysia, Myanmar, New Zealand, and the Philippines: *n* = 1, 2.1%). ‘Australian’ was the most (*n* = 26, 54.2%) reported ethnic background, followed by Italian and Indian (both: *n* = 2, 4.2%).

Children of respondents that underwent a SACS-R assessment by a MCH nurse were mostly male (*n* = 32, 68.1%), with an age range of 0.99 to 4.19 years (mean *=* 1.98; SD = 0.86). Almost all (*n* = 46, 97.9%) children were reported as being born in Australia. Most (*n* = 39, 83.0%) children were born at term, with 8.5% (*n* = 4) of children conceived with the assistance of IVF. Over half (*n* = 25, 53.2%) of the children had siblings; of these, most had one sibling (*n* = 19, 76.0%), and half (*n* = 12, 50.0%) of the siblings were female.

English was the most commonly spoken language at home (*n* = 46, 95.8%), with all other reported languages spoken at home equally represented (Filipino, Greek, Karenni, Malayalam, Mandarin, Portuguese, Punjabi, and Urdu: *n* = 1, 2.1%). In most cases the primary and secondary caregivers were married (*n* = 43, 89.6%), and the SACS-R assessed child lived with both the primary and secondary caregivers (*n* = 44, 91.7%). Annual family incomes varied, though the most commonly reported annual income brackets were ‘AUD$115,001 to AUD $135,000’ (*n* = 9, 19.1%) and ‘over AUD $175,000’ (*n* = 8, 17.0%).

The KAS appointment most participants had attended prior to completing the survey was the 18-month KAS (*n* = 18, 37.5%), with the 12-month KAS and the 24-month KAS consultations accounting for 27.1% (*n* = 13) and 25% (*n* = 12) of participants, respectively. Another type of appointment was attended by 10.4% (*n* = 5) of participants. Participants were asked if MCH nurse identified their child with a high likelihood for autism during the appointment, with 10.4% (*n* = 5) responding yes.

All parents/caregivers were asked for their level of agreement with a series of statements regarding their satisfaction with the SACS-R assessment, and their responses were predominantly positive. The majority of parents/caregivers were satisfied with the SACS-R process (*n* = 26, 72.2%), would recommend the SACS-R to other parents/caregivers (*n* = 24, 64.9%), and agreed that it was worthwhile assessing their child’s likelihood for autism (*n* = 33, 86.8%). Most also thought that the SACS-R was useful for all parents/caregivers of young children, not just those with previous concerns for their child (*n* = 30, 78.9%). Around half of respondents also reported that they now knew more about social attention and communication milestones in autism (*n* = 20, 52.6%) and in young children in general (*n =* 21, 55.3%) following their child undergoing a SACS-R assessment. When asked about their confidence in their MCH nurse’s ability to assess their child’s social communication behaviors, almost all felt confident (*n* = 35, 92.1%). Importantly, the majority of participants (*n* = 29, 76.3%) reported that they did not feel overwhelmed when having their child’s behavior assessed using MoSAIC. While just over half (*n* = 20, 55.6%) responded that they were satisfied with the help/support offered by their MCH nurse after receiving the SACS-R results, the remaining participants (*n* = 16, 44.4%) responded that they neither agreed nor disagreed, with none disagreeing.

## 4. Discussion

The early identification and support of Autistic infants and children is critical as it can result in improved outcomes for children and families (10, 20, 39, 40). This paper describes how the study team designed the MoSAIC program to train the entire Victorian MCH workforce to: improve competency in the early identification of autism; use an autism specific early identification tool – the SACS-R; initiate conversations about autism screening and a child’s ‘likelihood’ for autism; and refer infants and children with a high likelihood of autism for supports, services, and further assessment. To our knowledge this is the first study to demonstrate the feasibility of designing and implementing a very large-scale, state-wide, training program for a MCH nurse population serving a state with 6.66 million residents (41), or indeed any primary care population. The method included four key stages: (1) a training needs analysis, (2) training design, (3) training implementation, and (4) training evaluation. The results and findings of each stage have contributed to the existing literature on early autism training, identification, and referral for further assessment and access to services, as discussed below.

The training needs analysis with key stakeholders, which included four focus groups, a referral pathways workshop, and a training needs survey, collectively identified key findings relating to experiences of MCH nurses and the parents/caregivers who interfaced with them, as well as learnings from Autistic adults. Results indicated that parents/caregivers had mixed experiences regarding their relationships with MCH nurses, in line with previous research findings (42, 43). MCH nurses, parents/caregivers and Autistic adults agreed on the importance of early screening for autism, with Autistic adults highlighting the importance of this in the context of avoiding poor mental health outcomes associated with late diagnosis or misdiagnosis (44–46). The groups also expressed a view that it was important to normalize the introduction of the SACS-R screening alongside other well-baby checks, with Autistic adults and parents/caregivers voicing hope that this would also improve understanding of autism and reduce stigma within the community. The potential that some parents/caregivers may experience feelings of stress and/or stigma associated with participating in the check was also highlighted (47–49), particularly if their child was identified as having a high likelihood of autism (50, 51), which is consistent with previous literature. Parents/caregivers of children previously identified with a higher likelihood of autism via a SACS-R assessment with an MCH nurse, reported that the SACS-R screening process was ‘easy’ and an important step in obtaining further support for their child. They also identified an opportunity for process improvement, suggesting that MCH nurses proactively follow up with families after communicating a high likelihood result. MCH nurses reported experiencing difficulties with their local referral pathway, particularly with inconsistencies in access to services between areas, and in ensuring children with a high likelihood of autism received referrals to appropriate diagnostic services. They also highlighted that families often faced long waiting lists and/or high costs to access diagnosis and services for their child, consistent with findings from other studies (52, 53). The training needs survey also identified that most MCH nurses had not completed recent autism training and that many had gaps in current autism knowledge, which has been shown in other research with primary health providers in Australia, the UK, Canada, and the US (1, 54–56).

Based on the findings of the training needs analysis, a comprehensive autism training program (MoSAIC) was designed, along with supporting resources and pre-and post-training online modules. This comprehensive PDP was developed to provide the MCH workforce with appropriate training, while also including support resources to promote the retention of learning and to encourage the use of these learnings within their practice. High levels of training workshop attendance, both in-person and online, and completion of the pre-and/or post-workshop modules were attained by the project.

The training evaluation showed that the MCH workforce had a high level of satisfaction with the MoSAIC training. This may be explained by the MoSAIC training being designed specifically for the MCH workforce based on learnings from the training needs analysis. These results complement other literature evaluating autism training quality (57). The evaluation of the PDP also indicated that following the MoSAIC training, MCH nurses felt confident in using the SACS-R tool in their practice. This supports Barbaro’s (21) finding that 99% of the MCH nurses surveyed immediately after SACS training felt able to use the SACS tool to monitor young children for the early signs of autism (21), Shretha’s (31) report of a statistically significant increase in confidence in monitoring for autism immediately following SACS training for Nepalese family and child health visitors, with similar findings for well-child/Tamariki Ora nurses in New Zealand (32). The recent MCH workforce training was also shown to have increased autism knowledge and confidence in monitoring infants and children for the signs of autism, however, increases were not always statistically significant. This could be explained by the MCH workforce being relatively knowledgeable in several areas regarding social attention and communication prior to the training, and that those who were more confident in their knowledge may have been more likely to complete the training needs survey.

While this study was successful in demonstrating that a tailored early autism identification training program can be developed and successfully implemented, the study was not without its limitations. Given the relatively low sample sizes obtained, the results from the MCH workforce regarding training and implementation feedback, and from parents/caregivers on the training evaluation must be viewed with some caution. Optimally, gaining stakeholder feedback from a larger number of parents/caregivers and Autistic adults, and ensuring recruitment of First Nations people would have been ideal. Future research and PDP development should consider the fine balance between completion of such a project in a short period of time and sufficient time to fully engage with stakeholders representative of the whole community.

Overall, this study has demonstrated that it is possible to successfully design and implement a large-scale early social-communication and autism training program for primary healthcare professionals. The outcomes have demonstrated that a broad training needs analysis, with feedback from a wide variety of key stakeholders, can ensure that a PDP can be designed to meet the needs of professionals, parents/caregivers, and their Autistic children. It has also demonstrated that universal developmental monitoring of autism in the community is feasible and acceptable by all key stakeholders, including professionals, parents/caregivers, and Autistic adults, and should therefore be part of standard practice for all primary healthcare professionals.

## Data availability statement

The datasets presented in this article are not readily available due to contractual obligations with the study funder. Requests to access the datasets should be directed to JB, j.barbaro@latrobe.edu.au.

## Ethics statement

The studies involving human participants were reviewed and approved by La Trobe University Human Ethics Committee and the Department of Education and Training, State Government of Victoria. The participants provided their written informed consent to participate in this study.

## Author contributions

JB and MG contributed to the conception and design of the study. MG, KG, and JB collected the data. MG and KG organized the database and performed the statistical analyses. MG wrote the first draft of the manuscript. All authors wrote sections of the manuscript, contributed to manuscript revision, editing, read, and approved the submitted version.

## Funding

This training program was funded by the Victorian State Government (2018).

## Conflict of interest

JB developed the intellectual property (IP) for the SACS-R tool; La Trobe University currently owns the IP. The IP has been licensed to the Victorian State Government, earning royalties for La Trobe University. Funds are partially distributed to JB for the background IP.

The remaining authors declare that the research was conducted in the absence of any commercial or financial relationships that could be construed as a potential conflict of interest.

## Publisher’s note

All claims expressed in this article are solely those of the authors and do not necessarily represent those of their affiliated organizations, or those of the publisher, the editors and the reviewers. Any product that may be evaluated in this article, or claim that may be made by its manufacturer, is not guaranteed or endorsed by the publisher.

## Footnotes

^1^While there is no single preferred term for all people on the autism spectrum, recent research about language preference has found that identity-first language (e.g., Autistic person) was preferred by Autistic adults (23, 24). Thus, this paper will use identity-first language.

^2^ https://www.health.vic.gov.au/publications/promoting-social-attention-interaction-and-communication-skills

^3^ asdetect.org
