# Supplementary material for: Development, delivery, and evaluation of a training program for the early identification of autism: Monitoring of Social Attention, Interaction, and Communication
Source: Front Neurol. 2023 Jul 7;14:1201265. doi: 10.3389/fneur.2023.1201265 (PMC10361691; doi:10.3389/fneur.2023.1201265)
Supplement: Supplementary file 1 [file Data_Sheet_1.docx]

Supplementary Material

# Metropolitan and rural/regional parents/caregivers and SACS-R parents/caregivers focus group moderator guide

| 1 | Introduction |
| --- | --- |
|  | *Overview of Focus Group Process and key points/guidelines:*   - Welcome and thanks for attending. - I would like to introduce myself [name], and introduce you to my colleague [name] who will be co-moderating and taking notes. - To reiterate the points in the information sheet:   - This discussion will take around 1 hour.   - We would like to hear about your experiences with MCH nurse checks, the SACS, and referral and intervention pathways.   - We may need to move the conversation along so we can cover all discussion items and hear from everyone here today.   - Others’ beliefs and opinions must be respected.   - The discussion and the names of other participants must not be discussed outside of this session.   - We will be audiotaping the discussion but no identifying information will be used anywhere.   - Please discuss any concerns you have with a member of the research team. - Do you have any questions about the focus group process before we start? - I would now like you to introduce yourselves and please share with the group, how old is your child and when were they diagnosed with autism? |
| 2. | **Focus Questions* - key questions are underlined** |
|  | **General experiences with your MCH nurse:**   - Can you please tell us about your experience of attending MCH checks with your child? - Had you raised any concerns of autism with your MCH nurse? If so, what was this experience like? - How did the MCH nurse raise concerns with you about your child’s development? - Has your MCH nurse built a relationship with you and your family? How important is the relationship between you and your MCH nurse? What impact would the relationship with the MCH nurse have on having them assess your child? - What role does your MCH nurse play in your and your child’s life? - Do you usually see the same MCH nurse? - How do you feel before/after your MCH visits? - Do you feel your MCH nurse can guide you through health/developmental challenges?   **Experiences with the SACS process:**   - Can you please tell us about your experience of going through the SACS process? How did you feel while the MCH nurse carried out the assessment?   *SUB QUESTIONS/PROMPT AS PART OF THIS THEME:*   - - Were you aware that your child was being screened for signs of autism?   - How was the SACS implemented with you and your child? Did the MCH nurse explain the screening process with you and what they were specifically screening for?   - What did the MCH nurse say upon completing the SACS process? How did you respond or feel? What would have helped at this time?   - Did the nurse use the word “autism”? Would you have preferred if they did or did not use this word?   - Had you heard of autism before? What did autism mean to you? What was your understanding of autism? - In hindsight, has your experience going through SACS lead to earlier diagnosis? What impact has this potentially had?   **What happened next - referral process:**   - Upon leaving the MCH nurse appointment, what happened next? - What was the referral process like for you and your family? What were the next steps in your journey? Was this process clear for you? Who led this process? - How much follow up was there from the MCH nurse during this process? Did this follow up meet your needs? What would have helped during this process? - How easy or hard was it for you to then get a formal diagnosis? - Were there any barriers that got in your way at this time? - What sort of services were made available to you and your family? - What resources did you access at this time, or in hindsight, would have been beneficial?   *QUESTIONS SPECIFIC FOR RURAL/REGIONAL FOCUS GROUP*   - Were these in your local area, or did you have to travel? What additional challenges/barriers do you face living in a rural/regional area?   **Recommendations for training:**  As you know, we are evaluating families’ experiences with SACS and their MCH nurse. This is to help us develop a SACS training package for MCH nurses, to assist parents of young children in Victoria. We would like you to think back on your experiences and our discussions today, and tell us what we can do to improve the process and experience for families.   - What can MCH nurses do to help parents cope and adjust? What can MCH nurses do to help families access services? - From your experiences with the MCH service, what are improvements that could be introduced? - What are the greatest needs faced by people following identification of developmental concerns? What did you need most at this time in your child’s life? - Were your needs addressed during this diagnostic process? Were your partner’s needs also addressed? - Looking back at your experience with the SACS process, do you have any recommendations or things you feel could have been handled differently? - Knowing that the SACS is now going to be rolled out across Victoria, what advice do you have for the MCH nurses? And what advice do you have for the training team? - Is there anything we have not asked about that you would like to share about your experience with SACS? Please feel free to share your thoughts. |
| 3. | **Conclusion** |
|  | - Summarise critical points and ask for clarification/confirmation of accuracy (here and throughout) as a validity check. - Reflect and comment on the outcomes of session. - Debrief as needed. - Thank participants for their contribution and remind them that if they have any questions, they can speak to the research team, or contact us using the details provided on the participant information statement. |

- *Focus groups by their nature are dependent on group interaction, rather than following a question-answer format. Therefore, the focus questions serve as a guide only. Group moderators are responsible for maintaining focus on the core aim, which is to better understand and learn from parents’ experiences of having their child screened with the SACS. Flexibility is required in order to explore issues not previously anticipated, allow for spontaneity, and to ensure all voices are heard.*

# Autistic adults focus group moderator guide

| 1 | Introduction |
| --- | --- |
|  | *Overview of Focus Group Process and key points/guidelines:*  *Explanation of SACS and MCH training*   - Welcome and thanks for attending. - I would like to introduce myself [name], and introduce you to my colleague [name] who will be co-moderating and taking notes. - Some important points about this focus group before we start:   - This discussion will take around 1½ hours.   - We would like to hear about your experiences with diagnosis, referral and support pathways, and your thoughts on the training package we are developing for Maternal and Child Health Nurses for the early identification of autism.   - We may need to move the conversation along so we can cover all discussion items and hear from everyone here today.   - Others’ beliefs and opinions must be respected.   - The discussion and the names of other participants must not be discussed outside of this session.   - We will be audiotaping the discussion but no identifying information will be used anywhere.   - Please discuss any concerns you have with a member of the research team.   - Use of language in the group (preferred language around autism/pronouns) - Do you have any questions about the focus group process before we start? - I would now like you to introduce yourselves to the group |
| 2. | **Focus Questions* - key questions are underlined** |
|  | **Recommendations for training:**  As you know, we are developing a SACS training package for MCH nurses, to assist parents of young children in Victoria. We would like you to think back on your experiences and our discussions today, and tell us what we can do to improve the process and experience for families.   - What is your opinion on the decision to roll-out SACS at a state-wide level?   - Prompt: potential benefits and challenges of doing SACS with all children - What advice do you have for our team who will be training the nurses? - How do you think nurses should talk to families about the SACS process? - What kind of impacts do you think earlier diagnosis would have had for you?   - Prompt: How about for your family? - What can MCH nurses do to help parents understand autism and help their child and family? What can nurses do to help families access services? - What are the greatest needs faced by people following identification of autism? What did you need most at this time? - Knowing that the SACS is now going to be rolled out across Victoria, what advice do you have for the MCH nurses? And what advice do you have for the training team?   **General experiences with diagnosis, and referral and intervention pathways:**   - Can you please tell us about your experience of diagnosis? - If you can remember, how did you feel after your diagnosis? - What has been your experience with accessing referrals and services? - Can you think of any particularly memorable experiences? [probe for best and worst] - Do think there have been barriers to accessing referrals and services for you?   **General:**   - Is there anything we have not asked about that you would like to share about your experiences? Please feel free to share your thoughts. |
| 3. | **Conclusion** |
|  | - Summarise critical points and ask for clarification/confirmation of accuracy (here and throughout) as a validity check. - Reflect and comment on the outcomes of session. - Debrief as needed. - Thank participants for their contribution and remind them that if they have any questions, they can speak to the research team, or contact us using the details provided on the participant information statement. |

- *Focus groups by their nature are dependent on group interaction, rather than following a question-answer format. Therefore, the focus questions serve as a guide only. Group moderators are responsible for maintaining focus on the core aim, which is to better understand and learn from adults with autism about the best ways to rollout SACS. Flexibility is required in order to explore issues not previously anticipated, allow for spontaneity, and to ensure all voices are heard.*
